# *Nocardia cyriacigeorgica* in a Mallard (*Anas platyrhynchos*) from Arizona, USA

**DOI:** 10.3390/pathogens14070698

**Published:** 2025-07-15

**Authors:** Susan Knowles, Brenda M. Berlowski-Zier, Anne Justice-Allen, Barbara L. Bodenstein, Jeffrey M. Lorch

**Affiliations:** 1 U.S. Geological Survey, 6006 Schroeder Road, Madison, WI 53711, USA; bberlowski-zier@usgs.gov (B.M.B.-Z.); bbodenstein@usgs.gov (B.L.B.); jlorch@usgs.gov (J.M.L.); 2 Arizona Game and Fish Department, 5000 W. Carefree Highway, Phoenix, AZ 85086, USA; ajusticeallen@azgfd.gov

**Keywords:** Actinomycete, Aves, *Anas platyrhynchos*, bacteria, granuloma, histology, *Nocardia cyriacigeorgica*, nocardiosis, pathology, PCR

## Abstract

*Nocardia* spp. are opportunistic pathogens of humans, domestic animals, and wildlife that can cause high levels of morbidity and mortality. Here, we present a unique case of nocardial airsacculitis in a free-ranging mallard (*Anas platyrhynchos*) from Arizona, USA, and compare it to the hosts, geographic distribution, diagnostic methodology, and infection site of known nocardiosis cases in birds. A gross necropsy, histopathology, and bacterial culture were performed. There were no gross findings associated with the nocardiosis. Histopathology showed multiple granulomas expanding the air sac with intralesional filamentous bacteria that were Grocott’s methenamine silver-positive, Fite–Faraco and Ziehl–Neelsen acid-fast, positive with the Periodic acid–Schiff reaction, and variably Gram-positive. The organism was isolated in culture and identified as *Nocardia cyriacigeorgica* based on the sequencing of a 463 bp portion of the 16S rRNA gene. While reports of nocardiosis in the class Aves are rare and some are possibly misdiagnosed due to limited diagnostics, cases are reported globally, sometimes resulting in epizootics. More information is needed to understand whether immunosuppression plays a role in disease development in birds. Known to be an emerging pathogen in humans, *N. cyriacigeorgica* can be considered as a differential diagnosis for pulmonary and potentially cutaneous or disseminated infections in birds.

## 1. Introduction

*Nocardia* spp. (class Actinomycetes, order Mycobacteriales, family Nocardiaceae) are aerobic, non-sporulating Gram-positive, variably acid-fast, branching filamentous bacterial rods found ubiquitously in soil, plant material, decaying vegetable matter, and water [1,2]. *Nocardia* spp. are found globally in varied ecosystems but occur more commonly in tropical and subtropical climates [3]. While the taxonomy continues to evolve, the “List of Prokaryotic Names with Standing in Nomenclature” lists 129 valid species that occur globally [4,5]. They are opportunistic pathogens that infect humans, domestic animals, and terrestrial and aquatic wildlife with varying clinical presentations [5,6,7,8]. While reports of nocardiosis in humans are rare, with an estimated 500–1000 cases in the United States every year [5], the effects are significant as nocardiosis causes high levels of morbidity and mortality, especially in the immunocompromised [3,9]. Transmission occurs primarily through inhalation, cutaneous wounds, or ingestion, with inhalation considered the most common route [5]. Nocardiosis can be primarily cutaneous from direct inoculation, pulmonary from inhalation, or disseminated, which results from the hematogenous spread of the infection [3].

The taxonomy of *Nocardia* has changed over time, and historic cases may have been misidentified [3]. Before molecular methods were available, diagnosis was performed by examining the phenotype and chemotaxonomy of cultured colonies and by microscopy using special histochemical stains [3]. *Nocardia* can be identified with Gram and Grocott’s methenamine silver stains or the Periodic acid–Schiff reaction and is also acid-fast, unlike *Actinomyces* [10]. The sequencing of the 16S rRNA gene is now the gold standard for the identification and phylogenetic analysis of *Nocardia* sp. isolates [3]. However, high sequence similarity among species can cause difficulties with identification using this method [11]. In such cases, multilocus sequence analysis may provide more accurate taxonomic delineations [11].

Reports of nocardiosis in captive birds are rare, and reports in wild birds are even rarer [1]. We report a unique case of airsacculitis in a free-ranging mallard (*Anas platyrhynchos*) caused by *Nocardia cyriacigeorgica*, a rare opportunistic pathogen, and review the limited cases of nocardiosis in the class Aves. We discuss which *Nocardia* spp. are known to infect birds, the hosts of these infections, the geographic distribution of cases, the diagnostic methodology, and infection sites associated with these cases and compare these findings to the current case.

## 2. Case Report

### 2.1. History

A 1090 g, free-ranging, subadult male mallard was found dead in June 2023 in an urban pond in Chandler, Arizona, USA. The mortality of adult ducks and ducklings began at the pond in mid-May, and at the time of submission, the number of confirmed deaths was 15. Clinical signs in this group of ducks included weakness and dyspnea with rapid progression to death. Environmental temperatures reached 37.8 °C, and botulism was the suspected cause of the mortality event. The carcasses of this mallard and two additional subadult male mallards were collected, stored chilled, and submitted to the U.S. Geological Survey’s National Wildlife Health Center (Madison, WI, USA) for necropsy.

### 2.2. Necropsy Findings

Epicardial fat stores were good, cervical and visceral fat stores were adequate, and subcutaneous fat stores were scant. The overall body condition score for the duck was 3/5 [12]. Multiple foci of reddening, the largest of which measured 1 × 0.4 cm, were observed on the webbing of the right foot. These foci might represent hyperemia or hemorrhage, but the lesions were not evaluated histologically. Pectoral muscle was mildly dry with an adhesive surface (indicative of dehydration) and mildly atrophied. Lungs were wet and diffusely light red. No gross lesions were noted in the air sacs at necropsy. Tissue samples collected for histopathology were fixed in 10% neutral buffered formalin for at least 24 h, trimmed, and placed in tissue cassettes. The two additional duck carcasses were in poor postmortem condition and unsuitable for further diagnostic evaluation.

### 2.3. Histopathological Findings

**Figure 1:**
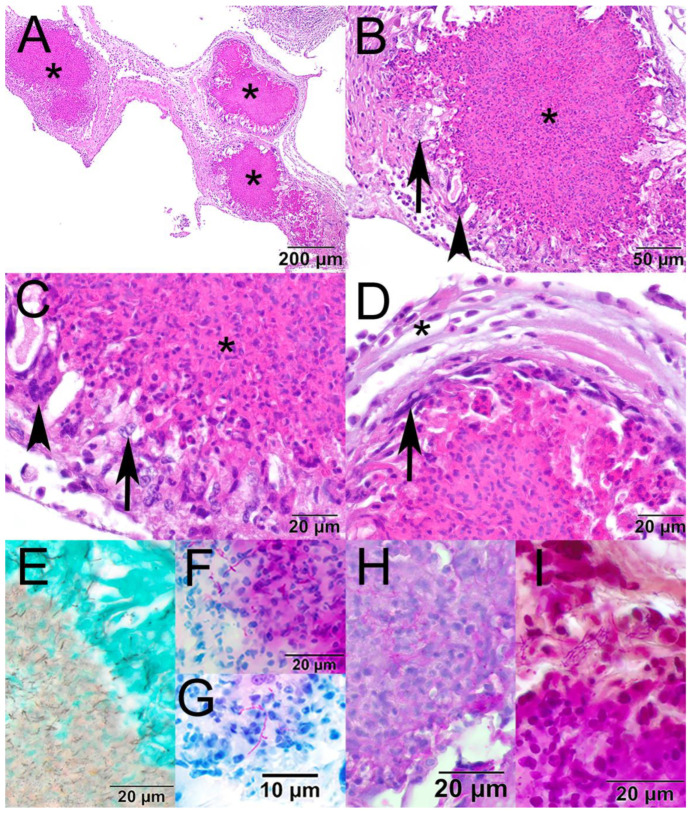
Photomicrographs from the air sac of a mallard (*Anas platyrhynchos*) found dead in Chandler, Arizona, USA. (**A**) The air sac is expanded by three large granulomas (asterisk; hematoxylin and eosin [H&E]). (**B**) The core of the granuloma is composed of eosinophilic and cellular debris (asterisk) and is surrounded by epithelioid macrophages (arrow) and multinucleated giant cells (arrowhead; H&E). (**C**) Higher magnification showing the core of the granuloma (asterisk), epithelioid macrophages (arrow), and a multinucleated giant cell (arrowhead; H&E). (**D**) Granulomas are occasionally surrounded by a thin rim of fibrous connective tissue (arrow) and further surrounded by low numbers of lymphocytes, plasma cells, and heterophils (asterisk; H&E). (**E**) The filamentous bacteria stained black with Grocott’s methenamine silver; (**F**) magenta with Fite–Faraco; (**G**) magenta with Ziehl–Neelsen; (**H**) magenta with the Periodic acid–Schiff reaction; (**I**) and variably basophilic and eosinophilic with a Brown and Hopps stain.

Fixed tissues were processed routinely, embedded in paraffin, sectioned at approximately 5 µm, stained with hematoxylin and eosin or Grocott’s methenamine silver, Fite–Faraco, Ziehl–Neelsen, the Periodic acid–Schiff reaction, and a Brown and Hopps at the Wisconsin Veterinary Diagnostic Laboratory (WVDL; Madison, WI, USA) [13], and examined with an Olympus BX43 microscope (Evident Scientific, Inc., Waltham, MA, USA). The examination of hematoxylin and eosin (H&E)-stained sections showed multiple granulomas expanding the air sac adjacent to the kidney (Figure 1A). Granulomas had a core of eosinophilic and cellular debris surrounded by epithelioid macrophages and fewer multinucleated giant cells (Figure 1B,C). Granulomas were occasionally surrounded by a thin layer of fibroblasts and further surrounded by low numbers of lymphocytes, plasma cells, and heterophils (Figure 1D). Filamentous bacteria within the granulomas were Grocott’s methenamine silver-positive (Figure 1E), Fite–Faraco acid-fast (Figure 1F), Ziehl–Neelsen acid-fast (Figure 1G), positive with the Periodic acid–Schiff reaction (Figure 1H), and variably Gram-positive with a Brown and Hopps stain (Figure 1I). Other histologic findings included rare perivascular cerebral hemorrhage, moderate pulmonary vascular congestion, and moderate splenic lymphoid depletion. A single trematode was present in the lumen of the cecum.

### 2.4. Diagnostic Findings

An air sac adjacent to the kidney was cultured on tryptic soy agar with 5% sheep blood at 37 °C and yielded the growth of a filamentous bacterium after 48 h of incubation. A portion of the 16S rDNA was amplified and sequenced as described previously from nucleic acid extracted from a pure isolate [14]. Based on a comparison of sequences available in GenBank, the 463 bp amplicon (with primer sequences removed) shared 100% sequence identity with the type strain of *N. cyriacigeorgica*. The next closest matches were to the type strains of *N. aurantiaca* (98.70%) and *N. kruczakiae* (98.49%). Sequence data generated from the isolate is available in GenBank under accession number PV535844. An avian influenza virus matrix reverse transcriptase polymerase chain reaction (RT-PCR) screen using tracheal and cloacal swabs was negative [15].

## 3. Discussion

Avian orders with known *Nocardia* spp. infections include Psittaciformes (*n* = 9) [1,16,17,18,19,20,21,22,23], Passeriformes (*n* = 4) [24,25,26,27], Galliformes (*n* = 2) [28,29], Accipitriformes (*n* = 1) [30], Anseriformes (*n* = 1) [31], Columbiformes (*n* = 1) [8], Gruiformes (*n* = 1) [32], and Procellariiformes [33] (*n* = 1). Prior to this report, nocardiosis was reported in only one Anseriformes individual, a captive domestic duck [31]. In the current case, nocardiosis was diagnosed in a sub-adult bird. Other reports do not show an age predilection for nocardiosis in birds, with ages of infected birds ranging from chicks to aged birds (Supplementary Table S1). While there was no evidence of immunosuppression in the reported cases, it is possible that some of the young birds were not fully immunocompetent [1,21,28], and that advanced age may have contributed to decreased immunocompetence [27]. Air pollution, overpopulation, and an underlying nutritional or metabolic defect were also suggested as potential predisposing causes in bird cases [8,17,32]. In the current case, there was splenic lymphoid depletion, which could indicate potential dysfunction in the immune response [34].

In the current case, 15 mallards were found dead, but only one was suitable for diagnostic evaluation, so it is unclear whether additional mallards from the event had nocardiosis. Cases in birds most often involve a single bird, but there are a few reported outbreaks of nocardiosis in commercial, captive, and wild birds. In one report from a commercial facility, mortality attributed to nocardiosis involved over 1000 turkey poults [28]. In captive birds, outbreaks involved 67 rock doves (*Columba livia*), 8 black crakes (*Zapornia flavirostra*), 2 Pesquet’s parrots (*Psittrichas fulgidus*), and 2 Moluccan king parrots (*Alisterus amboinensis hypophonius*) [8,17,18,32]. In wild birds, two Laysan albatrosses (*Phoebastria immutabilis*) from a colony were infected [33].

In birds, cases have been reported from Africa (*n* = 1) [30], Australia (*n* = 2) [21,22], Canada (*n* = 1) [16], China (*n* = 1) [1], Germany (*n* = 1) [25], India (*n* = 4) [8,23,26,31], Switzerland (*n* = 3) [18,20,32], and the United States (*n* = 6) [17,19,24,27,29,33] (Supplementary Table S1). In the United States, cases have been found in southern states including Georgia (*n* = 1) [17], Texas (*n* = 1) [24], and North Carolina (*n* = 1) [27] and on Midway Atoll in Hawaii (*n* = 1) [33]. As with our case from Arizona, in humans, most cases are found in the American southwest, where the climate is hot, dry, and windy [3].

From twenty reports of nocardiosis in birds (Supplementary Table S1), nine identified the *Nocardia* to the species level, with *N. asteroides* (*n* = 6) reported most frequently [18,20,24,25,29,33], followed by *N. nova* (*n* = 2) [1,32], and *N. farcinica/N. otitidiscaviarum* (*n* = 1) [28]. Here, we report an additional species, *N. cyriacigeorgica*, which we identified by 16S rRNA sequencing. Of the reported cases of nocardiosis in birds, only two were speciated using molecular methods [1,28]. It is important to note that seven cases were speciated by phenotypic testing only [18,20,24,25,29,32,33], and no species was provided for the remainder of the cases, in which bacteria were identified by histology alone (*n* = 6) [17,19,21,22,26,31], histology and in situ hybridization (*n* = 1) [27], and histology or cytology and culture (*n* = 4) [8,16,23,30].

In birds, there are reports of primary cutaneous (*n* = 1) [16], ocular (*n* = 1) [27], and pulmonary infections (*n* = 6) [19,20,23,31,32,33], as well as disseminated infections (*n* = 10) [1,8,17,18,21,22,24,25,26,28], caused by *Nocardia* spp. In humans, *N. cyriacigeorgica* can cause cutaneous, pulmonary, and disseminated infections [35,36]. In the current case, lesions caused by the N. *cyriacigeorgica* were mild and restricted to the air sac, and botulinum intoxication was the suspected cause of death.

## 4. Conclusions

While reports of nocardiosis in the class Aves are rare, cases are reported globally in eight orders of birds of various ages causing cutaneous, pulmonary, ocular, or disseminated infections. While most commonly affecting single birds, epizootics can occur. Known to primarily infect immunocompromised humans, the role of immunosuppression in the development of disease in birds is less well understood. Historic cases may have been misidentified, stressing the importance of sequencing and multilocus sequence analysis in the diagnostic process. While seemingly rare in birds, *Nocardia cyriacigeorgica* can be considered as a differential diagnosis for pulmonary and potentially cutaneous or disseminated infections with filamentous bacteria.

## Data Availability

Data for this study are available in a U.S. Geological Survey data release [37] at https://doi.org/10.5066/P1CG84WG.

## Supplementary Materials

The following supporting information can be downloaded at https://www.mdpi.com/article/10.3390/pathogens14070698/s1, Table S1: Cases of nocardiosis in Aves by genus and species, method of detection, host, age, and sex, location, commercial, captive or wild status, number affected, gross findings, and cytologic or histologic findings.
