# Supplementary material for: Nocardia cyriacigeorgica in a Mallard (Anas platyrhynchos) from Arizona, USA
Source: Pathogens. 2025 Jul 15;14(7):698. doi: 10.3390/pathogens14070698 (PMC12300570; doi:10.3390/pathogens14070698)
Supplement: Supplementary file 1 [file pathogens-14-00698-s001.zip › pathogens-3631185-supplementary.pdf]

Supplementary Table S1. Cases of nocardiosis in Aves by genus and species, method of detection, host, age, and sex, location, commercial, captive or wild status, number affected, gross findings, and cytologic or histologic findings.

**Genus and Species/Method of Detection**	**Host (Order)<br>Age<br>Sex**	**Location/<br>Commercial/<br>Captive or Wild/<br>Number affected**	**Gross Findings**	**Cytologic or Histologic Findings**	**Publication**
Nocardia cyriacigeorgica<br>Histology<br>Culture<br>PCR and sequencing	Mallard<br>*Anas platyrhynchos*<br>(Anseriformes)<br>Sub-adult<br>Male	Arizona, USA<br>Wild<br>*n*=1	No gross lesions observed in air sacs	Granulomas in air sac with GMS (+), F-F acid-fast, Z-N acid-fast, PAS (+), variably Gram (+) filamentous bacteria	This report
Nocardia farcinica or *Nocardia otitidiscaviarum*<br>Histology<br>Culture<br>PCR and sequencing	Domestic turkey<br>*Meleagris gallopavo*<br>(Galliformes)<br>5-day-old<br>NA	NA<br>Commercial<br>*n*=10<br>(mortality >1,000)	Multiple grey granulomas in congested lungs<br>Pale foci in kidneys<br>Hepatic and splenic congestion	Pulmonary and renal granulomas with variably Gram (+), variably Fite acid-fast filamentous bacteria	[1]
Nocardia nova<br>Histology<br>Culture<br>Multilocus sequencing	Yellow-bibbed lory<br>*Lorius chlorocercus*<br>(Psittaciformes)<br>45-day-old<br>NA	Hong Kong, China<br>Captive<br>*n*=1	Nodular, tan to yellow foci in lungs<br>Yellowish, firm material effacing sternal musculature	Granulomas in lung, spleen and sternal musculature with Gram (+), GMS (+), F-F acid-fast (not Z-N acid-fast) 0.5–1.0 µm wide, branching and beaded filamentous bacteria	[2]
Nocardia spp.<br>Histology	White-eyed conure<br>*Psittacara leucophthalmus*<br>(formerly *Aratinga leucophthalmus*)<br>(Psittaciformes)<br>9-year-old<br>Female	U.S.A.<br>Presumed Captive<br>*n*=1	NA	Abundant necrotic material and histiocytes in air sac with modified acid-fast bacteria	[3]
Nocardia spp.<br>Histology<br>In situ hybridization	Prothonotary warbler<br>*Protonotaria citrea*<br>(Passeriformes)<br>7-year-old<br>Female	North Carolina, U.S.A.<br>Captive<br>*n*=1	3-mm hard mass (inspissated exudate) on right eye<br>Swollen lower eyelid	Pyogranulomatous endophthalmitis with Gram (+), Fite acid-fast, and variably Z-N acid-fast bacteria<br>Retinal necrosis and detachment and posterior and anterior synechia	[4]
Suspect *Nocardia* spp. or *Actinomyces* spp.<br>Histology	Derbyan parrot<br>*Psittacula derbiana*<br>(Psittaciformes)<br>Nestling	New South Wales, Australia<br>Captive<br>*n*=1	Distinct, round 5–8 mm semi-firm nodules within and on the surface of the thigh musculature	Granulomas in lung, liver and kidney with Gram (+) (did not stain with Z-N or PAS)	[5]

	NA		Light-yellow, firm, discrete nodules in lungs, kidneys, and near the spine<br>Multiple foci of yellow discoloration in liver	branching, filamentous bacteria	
Nocardia spp.<br>Histology<br>Culture	Rock dove<br>*Columba livia*<br>(Columbiformes)<br>12–18-months-old<br>NA	Srinagar, India<br>Captive<br>*n*=67	Yellowish nodules in skin, lungs, air sacs, thoracic wall, pericardium, liver, joints and bones<br>Splenomegaly	Granulomas in various organs with Gram (+), Fite acid-fast (not Z-N acid-fast) filamentous, beaded, branching, bacteria	[6]
Nocardia nova<br>Histology<br>Culture	Black crake<br>*Zapornia flavirostra*<br>(formerly<br>*Limnocorax flavirostra*)<br>(Gruiformes)<br>Juvenile<br>NA	Basle, Switzerland<br>Captive<br>*n*=8	1–3 mm white, firm nodules throughout lungs<br>Splenomegaly	Pulmonary granulomas with Gram (+), F-F acid-fast (not Z-N acid-fast), 0.5–1.0 μm-wide, branching, occasionally beaded, filamentous bacteria	[7]
Nocardioform bacteria<br>Histology	Rainbow lorikeet<br>*Trichoglossus moluccanus*<br>(formerly<br>*Trichoglossus haematodus*)<br>(Psittaciformes)<br>4-year-old<br>NA	Perth, Australia<br>Captive<br>*n*=1	None	Necrotizing pectenitis with Giemsa (+) and Gram (+) (did not stain with modified Z-N) filamentous, branching 0.5–1.0 μm bacteria<br>Focal retinitis<br>Pulmonary and renal granulomas with filamentous bacteria	[8]
Nocardia asteroides<br>Cytology<br>Culture	Red-lored Amazon parrot<br>*Amazona autumnalis autumnalis*<br>(Psittaciformes)<br>15-year-old<br>Female	Zurich, Switzerland<br>Captive<br>*n*=1	Massive swelling of infraorbital sinuses with gray-green exudate	Aspirated fluid showed Gram (+), Z-N acid-fast, long, branching, filamentous bacteria	[9]
Nocardia spp.<br>Cytology<br>Histology<br>Culture	Hyacinth macaw<br>*Anodorhynchus hyacinthinus*<br>(Psittaciformes)<br>>20-year-old<br>Female	Canada<br>Captive<br>*n*=1	Multiple caseous nodules in pectoral muscle	Smears of exudate showed Gram (+), acid-fast filamentous bacteria<br>Histology showed granulomatous inflammation with acid-fast bacteria	[10]
Nocardia asteroides<br>Histology<br>Culture	Laysan albatross<br>*Phoebastria immutabilis*	Midway Atoll, Hawaii, U.S.A.<br>Wild<br>*n*=2	1 mm fibrinous plaques or fibrinous exudate adhered to air sac	Fibrinous airsacculitis with basophilic, branching, filamentous bacteria	[11]

	(formerly *Diomedea immutabilis*)<br>(Procellariiformes)<br>~4-month-old<br>NA				
Nocardia sp.<br>Cytology<br>Culture	Grey parrot<br>*Psittacus erithacus*<br>(Psittaciformes)<br>NA<br>NA	India<br>Captive<br>*n*=1	Pulmonary abscesses	Impression smear showed Gram (+) fragmented branched filaments in lung	[12]
Nocardia asteroides<br>Histology<br>Culture	Purple throated sunbird<br>*Leptocoma sperata*<br>(formerly *Nectarinia sperata*)<br>(Passeriformes)<br>NA<br>NA	San Antonio, Texas, U.S.A.<br>Captive<br>*n*=1	Diffuse red and pink mottling of lungs	Pulmonary granulomas with delicate Gram (+), F-F acid-fast (not Z-N acid-fast) 0.5–1 μm branched, occasionally beaded, filamentous organisms<br>Similar granulomas in heart and kidneys	[13]
Nocardia spp.<br>Histology	Pesquet's parrot<br>*Psittrichas fulgidus*<br>(Psittaciformes)<br>6-week-old<br>NA	Georgia, U.S.A.<br>Captive<br>*n*=2	Caseous necrosis in lungs and several small, white, raised foci on soft palate	Pulmonary (hepatic and splenic in one case) granulomas with modified Kinyoun's acid-fast, Gram (+), methenamine silver (+), branching, filamentous bacteria	[14]
Nocardia asteroides<br>Culture	Chicken<br>*Gallus gallus domesticus*<br>(Galliformes)<br>NA<br>NA	U.S.A.	NA	NA	[15]
Suspect *Nocardia asteroides*<br>Histology<br>Culture	Moluccan king parrot (paper refers to as blue-winged royal budgerigars)<br>*Alisterus amboinensis hypophonius*<br>(Psittaciformes)<br>NA<br>NA	Zurich, Switzerland<br>Captive<br>*n*=2	Pinpoint foci in lungs<br>Thick, dry crumbly, fibrin masses in air sac<br>Pea-sized, hard, necrotic lesion in kidney<br>Pinpoint necrotic foci in pectoral muscle	Pulmonary necrosis and granulomas, fibrinous air sacculitis and necrotizing arteritis with thrombosis with Grocott (+), PAS (+), Gram (+), F-F acid-fast (not Z-N acid-fast) long, slender, branching bacteria<br>Similar findings in gastric mucosa	[16]
Nocardia spp.<br>Presumed histology and culture	African fish eagle<br>*Icthyophaga vocifer*<br>(formerly *Haliaeetus vocifer*)<br>(Accipitriformes)	East Africa<br>Wild<br>*n*=1	NA	NA	[17]

	NA<br>NA				
Nocardia asteroides<br>Histology<br>Culture	Red-legged honeycreeper<br>*Cyanerpes cyaneus*<br>(Passeriformes)<br>NA<br>NA	Germany<br>Captive<br>*n*=1	Miliary yellow-gray foci in lungs, liver and kidneys<br>Splenomegaly	Pulmonary, hepatic and renal granulomas with long, thin, branched Z-N acid-fast, PAS positive bacteria	[18]
Nocardia spp.<br>Histology	Common hill mynah<br>*Gracula religiosa*<br>(Passeriformes)<br>8-month-old<br>NA	Odisha (formerly Orissa), India<br>Captive<br>*n*=1	Greyish white 2–8 mm nodules in lungs, kidneys, proventriculus, mesentery, and muscle of eye	Granulomas in multiple organs with Gram (+) and PAS (+) (not Z-N or F-F acid-fast) 0.5–0.8 μm filamentous and beaded bacteria	[19]
Nocardia spp.<br>Histology	Domestic duck<br>*Anas platyrhynchos*<br>(formerly *Anas boscas*)<br>(Anseriformes)<br>NA<br>NA	Hyderabad, India<br>Captive<br>*n*=1	Nodular 1–2 mm greyish-white lesions in lungs that sometimes coalesced	Pulmonary granulomas with 0.3–0.5 μm closely interwoven long thin branching, beaded variably Gram (+), PAS (+) (not Z-N acid-fast or alcohol fast) bacteria	[20]

PCR = polymerase chain reaction; NA = not applicable; GMS = Grocott's methenamine silver; F-F = Fite-Faraco; Z-N = Ziehl-Neelsen; PAS = periodic acid-Schiff reaction

1. Gazdzinski, P. Pulmonary Nocardiosis in turkey poults. *Zootecnica International* 2020, pp. 50–53.
2. Churgin, S.M.; Teng, J.L.L.; Ho, J.H.P.; Graydon, R.; Martelli, P.; Lee, F.K.; Hui, S.W.; Fong, J.Y.H.; Lau, S.K.P.; Woo, P.C.Y. First case report of fatal *Nocardia nova* infection in yellow-bibbed lory (*Lorius chlorocercus*) identified by multilocus sequencing. *BMC Vet Res.* **2019**, *15*, 4, doi:10.1186/s12917-018-1764-x.
3. Nemeth, N.M.; Gonzalez-Astudillo, V.; Oesterle, P.T.; Howerth, E.W. A 5-Year retrospective review of avian diseases diagnosed at the Department of Pathology, University of Georgia. *J Comp Pathol.* **2016**, *155*, 105–120, doi:10.1016/j.jcpa.2016.05.006.
4. Reynolds, T.L.; Barnes, H.J.; Wolfe, B.; Lu, L.; Camp, D.M.; Malarkey, D.E. Bilateral nocardial endophthalmitis in a prothonotary warbler (*Protonotaria citrea*). *Vet Pathol.* **2009**, *46*, 120–123, doi:10.1354/vp.46-1-120.
5. Park, F.J.; Jaensch, S. Unusual multifocal granulomatous disease caused by actinomycetous bacteria in a nestling Derbyan parrot (*Psittacula derbiana*). *Aust Vet J.* **2009**, *87*, 63–65, doi:10.1111/j.1751-0813.2008.00378.x.
6. Darzi, M.M.; Mir, M.S.; Nashiruddullah, N.; Kamil, S.A. Nocardiosis in domestic pigeons (*Columba livia*). *Vet Rec.* **2006**, *158*, 834–836, doi:10.1136/vr.158.24.834.
7. Bacciarini, L.N.; Posthaus, H.; Pagan, O.; Miserez, R. *Nocardia nova* causing pulmonary nocardiosis of black crakes (*Limnocorax flavirostra*). *Vet Pathol.* **1999**, *36*, 345–347, doi:10.1354/vp.36-4-345.
8. Raidal, S.R. Bilateral necrotizing pectenitis causing blindness in a rainbow lorikeet (*Trichoglossus haematodus*). *Avian Pathol.* **1997**, *26*, 871–876, doi:10.1080/03079459708419261.
9. Baumgartner, R.; Hoop, R.K.; Widmer, R. Atypical nocardiosis in a red-lored Amazon parrot (*Amazona autumnalis autumnalis*). *J Assoc Avian Vet.* **1994**, *8*, 125–127, doi:10.2307/30135067.

10. Breadner, S. Chronic *Nocardia* infection in a hyacinth macaw. In Proceedings of the Annual Conference, Association of Avian Veterinarians, Reno, Nevada, September 28–30, 1994; pp. 283–286.
11. Sileo, L.; Sievert, P.R.; Samuel, M.D. Causes of mortality of albatross chicks at Midway Atoll. *J Wildl Dis.* **1990**, *26*, 329–338, doi:10.7589/0090-3558-26.3.329.
12. Pal, M. Spontaneous nocardial infection in a grey parrot (*Psittacus erithaagus*). *Indian Vet Med J.* **1988**, *12*, 252–254.
13. Parnell, M.J.; Hubbard, G.B.; Fletcher, K.C.; Schmidt, R.E. *Nocardia asteroides* infection in a purple throated sunbird (*Nectarinia sperapa*). *Vet Pathol.* **1983**, *20*, 497–500, doi:10.1177/030098588302000414.
14. Long, P.; Choi, G.; Silberman, M. Nocardiosis in two Pesquet's parrots (*Psittrichas fulgidus*). *Avian Dis.* **1983**, *27*, 855–859.
15. Pier, A.C.; Fichtner, R.E. Distribution of serotypes of *Nocardia asteroides* from animal, human, and environmental sources. *J Clin Microbiol.* **1981**, *13*, 548–553, doi:10.1128/jcm.13.3.548-553.1981.
16. Ehrsam, v.H.; Hauser, B. Nocardiose bei blaufliigel-konigssittichen (*Alisterus amboinensis hypophonius*). *Schweiz Arch Tierheilk.* **1979**, *121*, 195–200.
17. Cooper, J.E. Post-mortem findings in East African birds of prey. *J Wildl Dis.* **1973**, *9*, 368–375, doi:10.7589/0090-3558-9.4.368.
18. Bergmann, A.; Schueppel, K.E.; Kronberger, H. Nocardiose bei einem turkisvogel (*Cyanerpes cyaneus*). In Proceedings of the Verhandlungsbericht des XV. Internationalen Symposiums über die Erkrankungen der Zootiere, Kolmarden, vom 27. Juni bis 1. Juli, 1973; pp. 293–296.
19. Iyer, P.K.; Rao, A.T.; Acharjyo, L.N.; Sahu, S.; Mishra, S.K. Systemic nocardiosis in a hill mynah (*Gracula religiosa*). A pathological study. *Mycopathol Mycol Appl.* **1972**, *48*, 223–229, doi:10.1007/BF02063061.
20. Iyer, P.K.; Rao, P.P. Suspected pulmonary nocardiosis in a duck. *Sabouraudia.* **1971**, *9*, 79–80, doi:10.1080/00362177185190201.
